# Cross-cultural validation of the sports performance questionnaire in Chinese adolescent football players: a confirmatory factor analysis study

**DOI:** 10.3389/fpsyg.2026.1837560

**Published:** 2026-07-23

**Authors:** Ying Shuai, Garry Kuan, Yee Cheng Kueh, Shaoshen Wang, Yan Sun

**Affiliations:** 1 Shandong Sport University, Jinan, Shandong, China; 2 Sports Social Science Research Institute, Shandong Sport University, Jinan, Shandong, China; 3 Biostatistics and Research Methodology Unit, School of Medical Sciences. Universiti Sains Malaysia, Kubang Kerian, Kelantan, Malaysia; 4 Exercise and Sports Science Programme, School of Health Sciences, Universiti Sains Malaysia, Kubang Kerian, Kelantan, Malaysia; 5 School of Economics and Management, Shanghai University of Sport, Shanghai, China

**Keywords:** Chinese adolescents, confirmatory factor analysis, cross-cultural validation, sports performance assessment, talent identification, youth football

## Abstract

**Background:**

The assessment of sports performance in youth football requires culturally appropriate and psychometrically sound instruments. The Sports Performance Questionnaire (SPQ) was developed to evaluate performance-related characteristics in youth football players, but its cross-cultural validity in Chinese contexts remains unexplored.

**Objective:**

This study aimed to validate the Chinese adaptation of the Sports Performance Questionnaire (SPQ-C) among adolescent football players and examine its factorial structure, reliability, and validity.

**Methods:**

A cross-sectional validation study was conducted with 450 adolescent football players (52.2% male, mean age = 12.94 ± 0.78 years) from 24 schools across urban areas of Shandong Province, China. The SPQ-C was administered as a coach-rating instrument, consistent with the format of the original SPQ. Confirmatory factor analysis was used to test the original two-factor structure and a unidimensional model. Reliability and validity were assessed using established psychometric criteria.

**Results:**

The original two-factor model showed broadly adequate global fit; however, it was not retained because the correlation between the Persistence and Skill factors was extremely high (r = 0.996), indicating empirical indistinguishability and a lack of discriminant validity. The unidimensional model was therefore retained as the final SPQ-C structure. The final model showed adequate global fit, although the RMSEA value was slightly above the stringent good-fit threshold. The SPQ-C demonstrated excellent internal consistency (*α* = 0.932), satisfactory convergent validity (AVE = 0.559, CR = 0.932), and high preliminary test–retest reliability in a single-school subsample (ICC = 0.935). Standardized factor loadings ranged from 0.609 to 0.841.

**Conclusion:**

The SPQ-C provides preliminary evidence of reliability and validity as a unidimensional coach-rated instrument for assessing performance-related characteristics in Chinese adolescent football players from urban areas of Shandong Province. The findings suggest that, in the present Chinese coach-rating context, persistence- and skill-related indicators were not empirically separable. Further validation is needed in more geographically diverse samples and with larger multi-school test–retest subsamples.

## Introduction

Sports performance is a multifaceted construct that reflects the integration of physical capacity, technical skill, tactical understanding, psychological attributes, and behavioral consistency in sport-specific contexts (D’Isanto et al., 2019; Kanosue et al., 2015). In youth football, performance evaluation cannot be reduced to objective indicators such as running distance, speed, or match statistics alone, because coaches also need to assess non-physical and context-dependent characteristics, including competitive behavior under pressure, tactical discipline, communication, cooperation, self-regulation, and creativity (Allen et al., 2013; Bergkamp et al., 2019; Musculus and Lobinger, 2018). These characteristics are particularly important in adolescent football, where performance is closely linked to ongoing skill acquisition, psychological development, and adaptation to team-based competitive environments.

Although electronic performance and tracking systems provide valuable objective information about physical load and match behavior, they do not fully capture coach-perceived psychological, tactical, and behavioral dimensions of performance (Cossich et al., 2023; González-Víllora et al., 2015). Therefore, standardized coach-assessment instruments remain necessary as complementary tools for evaluating performance-related characteristics that are not easily captured through objective metrics alone.

The assessment of sports performance has evolved significantly, moving beyond simple elite versus non-elite categorizations toward more nuanced measurement approaches that account for the multifactorial nature of athletic achievement (Swann et al., 2015). This evolution is particularly relevant in football, where success encompasses not only competitive outcomes but also the development of technical skills, tactical awareness, and psychological resilience across different performance levels (Allen et al., 2013; Bergkamp et al., 2019). Contemporary performance analysis emphasizes the systematic process of evaluating and enhancing player performance through the collection, examination, and interpretation of data from multiple sources. Modern approaches integrate video analysis, physical performance metrics, and psychological assessment to provide comprehensive insights into player development trajectories (Cossich et al., 2023).

### Challenges in football performance measurement

Traditional approaches to evaluating football performance have predominantly relied on ordinal classifications based on competition level, team selection status, and achievement milestones (McAuley et al., 2022). However, these methods often fail to capture the nuanced aspects of player development and performance variability across different contexts (Lepschy et al., 2018). The complexity of football performance assessment is further compounded by the sport’s inherent demands for integration across multiple domains, including physical capabilities, technical proficiency, tactical understanding, and psychological attributes (Bonney et al., 2019).

The limitations of existing measurement approaches have prompted researchers to develop more comprehensive assessment tools that can adequately capture the multidimensional nature of football performance. This need is particularly acute in youth football, where performance evaluation must account for developmental trajectories and the dynamic nature of skill acquisition (Musculus and Lobinger, 2018).

### The sports performance questionnaire (SPQ)

In response to these measurement challenges, Sivrić (2021) developed the Sports Performance Questionnaire (SPQ), a comprehensive instrument designed to assess success factors in youth football. The SPQ was developed through rigorous methodological procedures, including expert consultation with football coaches and focus group discussions to identify key performance indicators relevant to youth football success. The instrument encompasses multiple dimensions of performance, including competitiveness under pressure, on-field confidence, teamwork and communication, self-evaluation capabilities, pressure management, discipline, adherence to tactical requirements, and creativity.

The development of SPQ represents a significant advancement in football performance measurement, as it addresses the identified gaps in existing assessment tools by providing a standardized, multidimensional approach to evaluating youth football performance. The theoretical foundation of the SPQ draws from established models of athletic excellence in team sports (Trninić et al., 2010) while incorporating contemporary understanding of performance psychology and skill development.

### Cross-cultural validation in sports psychology

The development of reliable and valid psychological instruments for sports performance assessment has become increasingly important in contemporary sports science (Currell and Jeukendrup, 2008). Recent advances in sports psychology have emphasized the need for comprehensive measurement tools that can assess multiple dimensions of athletic performance across diverse cultural contexts (Wang et al., 2024). Cross-cultural validation of sports psychological instruments presents unique challenges, as cultural factors can significantly influence the interpretation and response patterns to performance-related questionnaires (Borsa et al., 2012).

The importance of rigorous cross-cultural validation procedures has been demonstrated across various sports psychology domains. For instance, the cross-cultural adaptation of the Sport Emotion Questionnaire across seven African countries revealed significant cultural variations in emotional expression patterns among athletes (Quansah et al., 2024). Similarly, the development and validation of the Sport Mental Training Questionnaire across Polish and English populations highlighted the necessity of examining factorial structures across different cultural contexts (Behnke et al., 2019). These studies underscore the critical need for systematic validation procedures when adapting psychological instruments for use in different cultural settings.

Recent research has also emphasized the importance of establishing measurement equivalence across cultures to ensure that psychological constructs are interpreted consistently across different populations (Rawat and Błachnio, 2022). The cross-cultural validation process typically involves multiple phases, including translation procedures, content validity assessment, and psychometric evaluation using confirmatory factor analysis (CFA) to establish structural validity (Arafat et al., 2016; Qi et al., 2025).

### Youth football development in China

The growing popularity of football in China and the increasing emphasis on youth development programs highlight the need for culturally appropriate assessment tools that can support talent identification and development processes. China’s commitment to football development is evidenced by the comprehensive national reform plan initiated in 2015, which outlined ambitious goals for youth football participation and talent development (General Office of the State Council, 2015). The reform established clear performance targets, including the participation of over 30 million school-age children in football by 2020 and the development of systematic talent identification and assessment mechanisms (Yang, 2025).

Recent research has identified significant challenges in Chinese youth football development, particularly regarding the lack of standardized assessment tools for evaluating player performance and development (Sarmento et al., 2018). Despite the establishment of over 32,000 football specialty schools across China, systematic evaluation methods for assessing youth football talent remain limited (Yang, 2025). The current talent development pathway in Chinese football is characterized by fragmented approaches across different governing bodies, creating challenges for consistent performance evaluation and talent identification (Gündoğan, 2024).

The establishment of scientifically validated assessment tools is particularly crucial given China’s unique cultural context and the specific characteristics of Chinese youth football development. Recent studies have highlighted the importance of developing culturally appropriate evaluation methods that can effectively assess both technical and psychological aspects of football performance in Chinese contexts (Wang et al., 2024). Furthermore, the integration of evidence-based assessment tools into Chinese youth football programs is essential for supporting the national goals of football development and talent cultivation (Yang, 2025).

### Study rationale and objectives

Despite the rapid development of youth football programs in China, there remains a lack of validated coach-assessment-based instruments for evaluating performance-related characteristics among Chinese adolescent football players (Wang et al., 2024; Yang, 2025). This gap is important because coaches are often responsible for identifying talent, monitoring player development, and making training decisions, yet they require standardized tools that are both psychometrically sound and culturally appropriate (Musculus and Lobinger, 2018). The SPQ provides a structured coach-rating framework for assessing key performance-related characteristics in youth football (Sivrić, 2021); however, its factorial structure and validity have not been examined in the Chinese context. Therefore, the present study aimed to evaluate the psychometric properties of the Chinese adaptation of the SPQ (SPQ-C) among adolescent football players and to determine whether the original two-factor structure or a unidimensional structure provides a more appropriate representation of coach-rated sports performance in this population.

The successful validation of the SPQ in Chinese youth football contexts would contribute significantly to the international standardization of performance assessment tools and provide practitioners with a culturally appropriate instrument for evaluating multiple dimensions of football performance. This research addresses a critical gap in the literature by providing empirical evidence for the cross-cultural validity of a comprehensive football performance assessment tool, while also supporting China’s national objectives for youth football development and talent identification. The findings will have important implications for sports psychology practitioners, youth football coaches, and talent development programs operating in Chinese contexts.

## Materials and methods

### Participants

A cross-sectional validation study design was implemented to examine the psychometric characteristics of the Chinese adaptation of the Sports Performance Questionnaire (SPQ-C). The study sample comprised adolescent football players between 12 and 15 years of age from urban areas of Shandong Province, China, who maintained active involvement in structured football training and competition through participating schools or football development programs. Therefore, the source population represented a defined urban school-based youth football context rather than all Chinese adolescent football players. Recruitment strategies focused on secondary educational institutions across 12 metropolitan areas within Shandong Province, with particular emphasis on schools demonstrating established football development programs and administrative support for research participation. This approach resulted in a source population representing a defined segment of the broader target demographic.

The sampling approach utilized comprehensive player registration databases maintained by participating in educational institutions, developed through collaborative efforts between school administrators and football coaching personnel to ensure complete coverage of all registered athletes meeting study requirements. This systematic approach facilitated accurate identification and recruitment of eligible participants while maintaining adherence to inclusion standards.

Inclusion parameters for study participation comprised: (1) Chinese citizenship; (2) chronological age between 12–15 years; (3) current enrollment in participating secondary schools within Shandong Province; (4) active membership in institutional football teams with minimum one-year training background; (5) adequate Chinese language proficiency and reading comprehension; (6) voluntary agreement to participate supported by both individual consent and guardian approval.

Sample size determination for confirmatory factor analysis procedures was conducted using Arifin’s web-based calculation platform, incorporating established guidelines from contemporary structural equation modeling research (Arifin, 2026). The computation integrated specific methodological parameters: projected Comparative Fit Index (CFI) value of 0.95, two-factor structure containing 8 indicators for the first factor and 3 indicators for the second factor, anticipated standardized factor loadings of 0.6, inter-construct correlations of 0.25, Type I error probability (*α*) set at 0.05 (bidirectional), and target statistical power (1 - *β*) of 90%. Following adjustment for anticipated study withdrawal (20%), the minimum sample requirement was established at 422 participants. The final achieved sample size (*N =* 450) surpassed this threshold, providing sufficient statistical power for reliable parameter estimation and factorial solution stability.

## Measures

### Sports performance questionnaire (SPQ)

The Sports Performance Questionnaire represents a coach-rating instrument developed to assess performance-related characteristics in youth football players, grounded in the Hypothetical Model of Specific Traits of Top Athletes in Team Sports Games (Trninić et al., 2010). This theoretical framework posits that specific personality attributes and psychological characteristics constitute fundamental determinants of success in team sport environments. The instrument was specifically designed to evaluate perceived sports performance dimensions among adolescent football players participating in competitive team sport contexts.

The SPQ consists of 11 items measuring performance-related characteristics across two originally proposed domains. Each item addresses a specific aspect of football performance, including competitive behavior in challenging situations, game assertiveness, motivation for personal development, collaborative skills, on-field communication effectiveness, self-evaluative capacity, stress management ability, self-regulatory discipline, adherence to tactical directives, natural ability, and creative expression. The original SPQ was designed as a coach-rating instrument, and this format was retained in the present Chinese adaptation (Sivrić, 2021). Thus, in this study, football coaches rated each athlete using a 5-point Likert-type response scale, ranging from 1 (completely absent) to 5 (fully demonstrated). This assessment approach enabled coach-based evaluation of psychological, behavioral, tactical, and skill-related characteristics relevant to youth football performance.

Psychometric evaluation of the Initial SPQ revealed a robust two-factor structure through exploratory factor analysis. The first factor, labeled “Persistence,” encompasses eight performance dimensions including improvement motivation, self-discipline, cooperative behavior, self-evaluation, competitive drive, tactical compliance, assertive play, and stress tolerance (*α* = 0.881). The second factor, designated “Skill,” incorporates three components: natural talent, creative abilities, and communication effectiveness (α = 0.827). The complete instrument demonstrated excellent internal consistency reliability (α = 0.904), supporting its utility for assessing perceived sports performance in team sport contexts (Sivrić, 2021).

### Cross-cultural adaptation

The SPQ was translated into Chinese following established guidelines for cross-cultural adaptation of psychological instruments (Beaton et al., 2000). The process involved forward translation by two independent bilingual experts (one in psychological assessment, one in sports psychology), synthesis of translations, back-translation by two independent translators blinded to the Initial version, and expert panel review for semantic and conceptual equivalence. Twenty youth football players (10 male, 10 female) participated in pilot testing to assess item clarity and cultural appropriateness. Final modifications were made based on their feedback, and professional proofreading ensured linguistic accuracy. This process maintained the instrument’s psychometric integrity while ensuring cultural relevance for Chinese youth football contexts.

### Ethics and procedures

This investigation obtained ethical clearance from the Universiti Sains Malaysia Human Research Ethics Committee (USM/JEPeM/22050288). The Chinese adaptation of the SPQ (SPQ-C) was conducted through systematic translation and validation procedures following internationally recognized cross-cultural adaptation protocols.

Data collection was conducted at participating schools between October and December 2023. After obtaining written parental consent and adolescent assent, player demographic information was verified through school/team registration records and participant documentation. The SPQ-C was then completed by football coaches who were familiar with the players’ training and match performance. Coaches rated each eligible player independently using the 11 SPQ-C indicators and the standardized 5-point response scale. Trained research personnel provided uniform instructions to coaches and were available to clarify administrative procedures, but they did not influence the ratings. Completed coach-rating forms were collected immediately and checked for completeness. A selected subsample of 50 players from Shandong Luneng Taishan Football School participated in retest administration after a two-week interval. The same coach-rating procedure was used at both time points. Because this subsample was drawn from a single elite football school, the retest analysis was intended to provide preliminary evidence of temporal stability rather than definitive evidence generalizable to all youth football settings.

### Statistical analysis

Initial data examination utilized SPSS 28.0 (IBM Corp, Armonk, NY, USA) to evaluate distributional characteristics and data quality. Univariate normality assessment employed Kolmogorov–Smirnov and Shapiro–Wilk tests, while multivariate normality was examined using Mardia’s coefficients for both skewness and kurtosis parameters (Ghasemi and Zahediasl, 2012). Distributional assessment was further supported through visual inspection of frequency histograms and chi-square versus Mahalanobis distance scatterplots, consistent with established analytical protocols (Tabachnick et al., 2019).

Confirmatory Factor Analysis (CFA) was conducted using Mplus 8.7 software with Maximum Likelihood Robust estimation procedures, chosen to accommodate potential non-normal distribution patterns as recommended in contemporary psychometric literature (Muthén and Muthén, 1998). Model fit evaluation utilized multiple goodness-of-fit indices with predetermined acceptability criteria: Comparative Fit Index (CFI > 0.95), Tucker-Lewis Index (TLI > 0.95), Root Mean Square Error of Approximation (RMSEA < 0.06, including 90% confidence intervals), and Standardized Root Mean Square Residual (Hu and Bentler, 1999). Factor loadings were deemed acceptable when exceeding 0.40, consistent with established psychometric standards (Hair et al., 2012).

Construct validity examination incorporated comprehensive convergent and discriminant validity analyses. Convergent validity verification involved assessment of standardized factor loadings (> 0.50), Average Variance Extracted (AVE > 0.50), and Composite Reliability indices (CR > 0.70), following recognized methodological frameworks (Hair et al., 2012). Discriminant validity was evaluated through the Fornell-Larcker criterion, requiring each construct’s AVE square root to surpass its inter-construct correlations (Fornell and Larcker, 1981).

Reliability evaluation encompassed internal consistency and temporal stability assessments. Internal consistency was determined through Composite Reliability coefficients, with values above 0.70 indicating adequate reliability (Taber, 2018). Test–retest reliability was analyzed using two-way mixed effects Intraclass Correlation Coefficients (ICC), with interpretation based on established benchmarks: insufficient (< 0.50), moderate (0.50–0.75), good (0.75–0.90), and excellent (> 0.90) reliability (Koo and Li, 2016).

## Results

### Descriptive statistics

Table 1 displays the descriptive characteristics of the confirmatory factor analysis sample (*N =* 450). Participant ages averaged 12.94 years (SD = 0.78), reflecting a predominantly early adolescent cohort. Gender representation was approximately equivalent, comprising 52.2% males (*n =* 235) and 47.8% females (*n =* 215). Recruitment encompassed 12 municipal areas throughout Shandong Province, with highest participation rates observed in Weifang (15.3%), Linyi (13.1%), and Zibo (10%). The cohort represented 24 distinct educational institutions, with Shandong Luneng Taishan Football School contributing the largest institutional representation (11.1%). Academic year distribution showed predominance of second-year junior secondary students (52.7%), with first-year students comprising 34.4% and third-year students representing 12.9% of the sample. Regarding their positions on the football field, the sample included a mix of defenders (39.3%), midfielders (26.2%), forwards (25.1%), and goalkeepers (9.3%).

**Table 1:** Demographic information and frequency of participants.

Category	Name	Frequency	Percent	Mean (SD)
Age				12.94(0.78)
City	Binzhou	19	4.2	
	Dongying	33	7.3	
	Heze	18	4.0	
	Jinan	37	8.2	
	Jining	28	6.2	
	Liaocheng	35	7.8	
	Linyi	59	13.1	
	Qingdao	41	9.1	
	Tai’an	38	8.4	
	Weifang	69	15.3	
	Zaozhuang	28	6.2	
	Zibo	45	10.0	
School	Binzhou Development Zone No.2 Middle School	19	4.2	
	Chengyang Experiment	19	4.2	
	Dianliu No.1 Middle School	19	4.2	
	Dongying Experiment	33	7.3	
	Heze Caozhou Military School	18	4.0	
	Jining University Affiliated Middle School	15	3.3	
	Liaocheng Shaolin	17	3.8	
	Linyi Phoenix Experiment	13	2.9	
	Linyi No.16 Middle School	14	3.1	
	Linzi Experiment	26	5.8	
	Shandong Luneng Taishan Football School	50	11.1	
	Qingdao Chengyang	22	4.9	
	Qingzhou Banner City	19	4.2	
	Shifeng Middle School	18	4.0	
	Tai’an Kaiyuan	18	4.0	
	Tai’an No.1 Middle School	20	4.4	
	Tancheng Nurturing Talents	18	4.0	
	Tangye Middle School	18	4.0	
	Wenshang Experiment	13	2.9	
	Yinan Huate Wolong	14	3.1	
	Zaozhuang No.15 Middle School	14	3.1	
	Zaozhuang Experiment	14	3.1	
	Zhangdian No.8 Middle School	13	2.9	
	Zibo No.5 Middle School	6	1.3	
Gender	Male	235	52.2	
	Female	215	47.8	
Grade	Grade 1 of junior high school	155	34.4	
	Grade 2 of junior high school	237	52.7	
	Grade 3 of junior high school	58	12.9	
Position	Forward	113	25.1	
	Midfielder	118	26.2	
	Defender	177	39.3	
	Goalkeeper	42	9.3	

SD, Standard Deviation.

Table 2 shows the score distribution for the 11 items of the SPQ-C (Chinese) scale. All items have mean scores above 3. The highest mean score was 4.16 (SD = 0.94) for SPQ3, with 81.8% of coach ratings falling in the “mostly demonstrated” or “fully demonstrated” categories. The lowest mean score was 3.43 (SD = 0.95) for SPQ10, with 44.9% of coach ratings falling in the “mostly demonstrated” or “fully demonstrated” categories and 42.2% in the “partially demonstrated” category. SPQ10 assessed talent, which may be a more inferential and context-dependent performance characteristic than more directly observable indicators such as cooperation, self-discipline, or tactical adherence. Therefore, coaches may have been more cautious when assigning high or low ratings on this item. Nevertheless, SPQ10 was retained because its standardized factor loading in the final unidimensional model was strong (0.777), indicating that it contributed meaningfully to the overall sport performance construct. Overall, the distribution of SPQ-C item scores suggested that coaches generally rated players’ performance-related characteristics positively.

**Table 2:** Distribution of item scores for the SPQ-C.

Items	1 *n* (%)	2 *n* (%)	3 *n* (%)	4 *n* (%)	5 *n* (%)	Mean (SD)
SPQ1	9 (2.0)	16 (3.6)	116 (25.8)	196 (43.6)	113 (25.1)	3.86 (0.90)
SPQ2	15 (3.3)	21 (4.7)	109 (24.2)	205 (45.6)	100 (22.2)	3.79 (0.95)
SPQ3	10 (2.2)	18 (4.0)	54 (12.0)	178 (39.6)	190 (42.2)	4.16 (0.94)
SPQ4	15 (3.3)	12 (2.7)	88 (19.6)	212 (47.1)	123 (27.3)	3.92 (0.93)
SPQ5	9 (2.0)	21 (4.7)	91 (20.2)	204 (45.3)	125 (27.8)	3.92 (0.92)
SPQ6	15 (3.3)	20 (4.4)	105 (23.3)	207 (46.0)	103 (22.9)	3.81 (0.95)
SPQ7	11 (2.4)	29 (6.4)	128 (28.4)	201 (44.7)	81 (18.0)	3.69 (0.92)
SPQ8	10 (2.2)	29 (6.4)	150 (33.3)	186 (41.3)	75 (16.7)	3.64 (0.91)
SPQ9	10 (2.2)	18 (4.0)	101 (22.4)	208 (46.2)	113 (25.1)	3.88 (0.91)
SPQ10	13 (2.9)	45 (10.0)	190 (42.2)	140 (31.1)	62 (13.8)	3.43 (0.95)
SPQ11	7 (1.6)	44 (9.8)	155 (34.4)	167 (37.1)	77 (17.1)	3.58 (0.94)

1 = completely absent; 2 = rarely demonstrated; 3 = partially demonstrated; 4 = mostly demonstrated; 5 = fully demonstrated; SD, standard deviation.

### Confirmatory factor analysis

**Figure 1:**
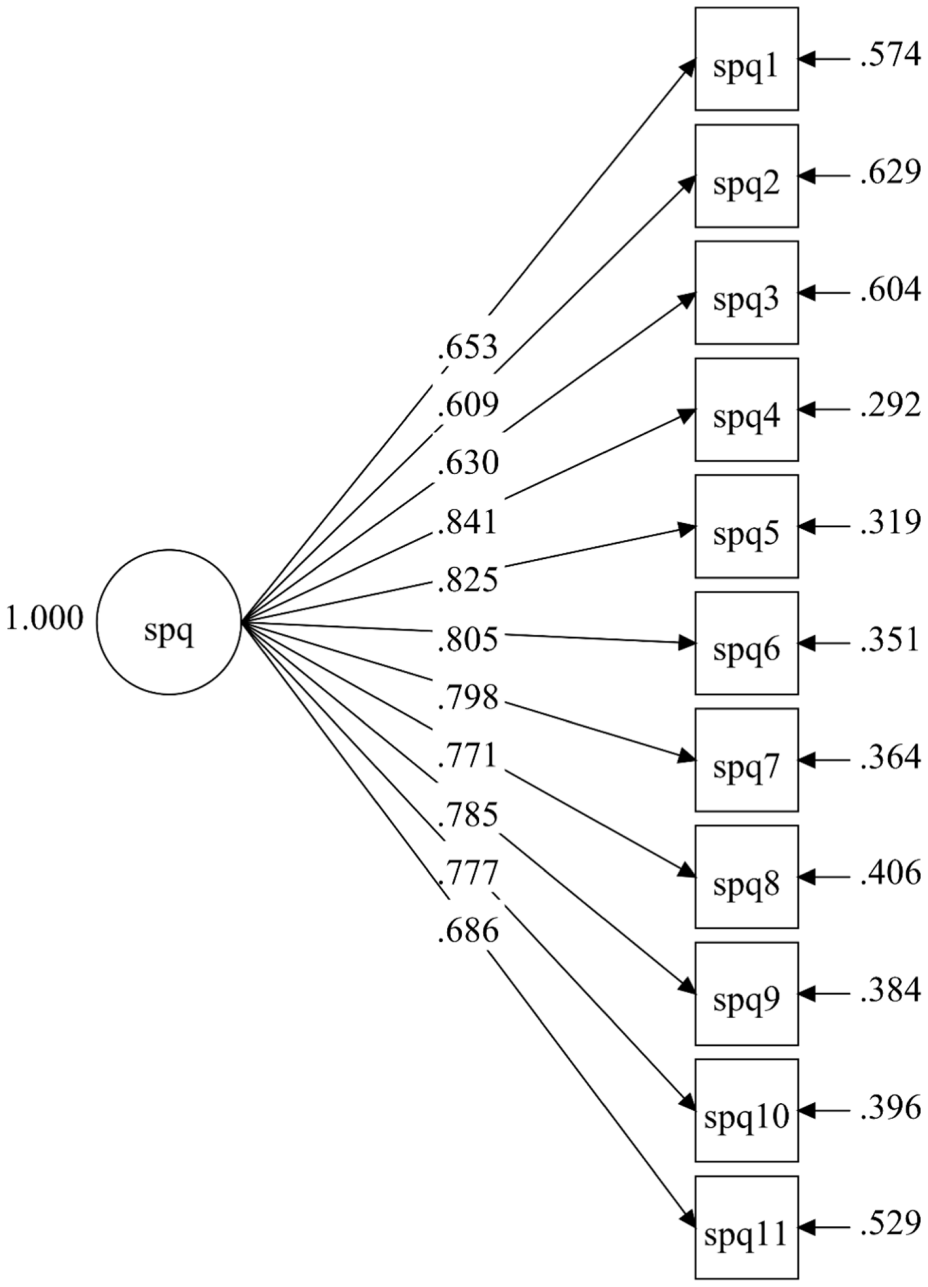
CFA diagram of the retained unidimensional SPQ-C model. SPQ1-SPQ11 = Sports Performance Questionnaire items 1–11; Sport Performance = unified sport performance construct.

**Figure 2:**
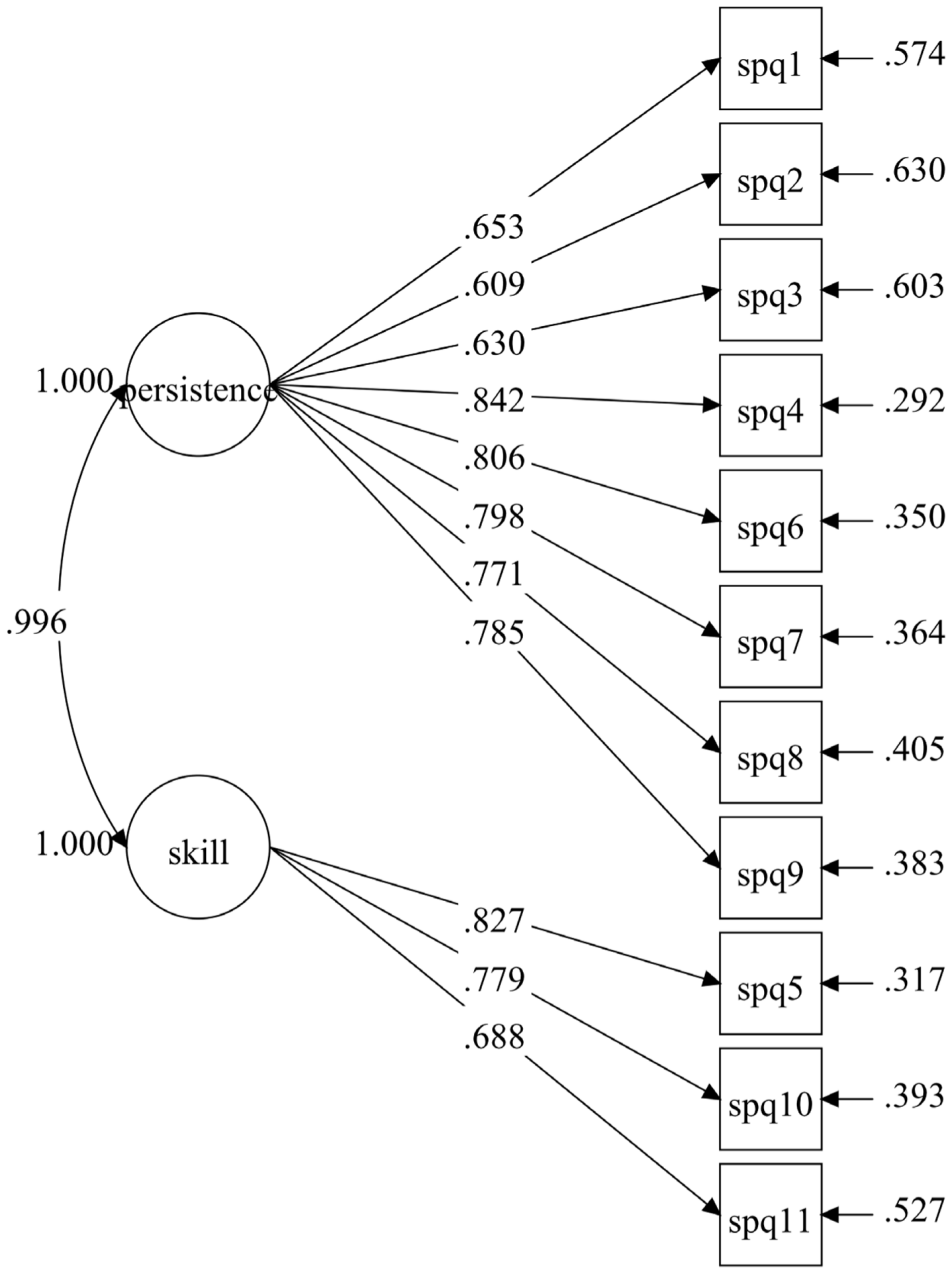
CFA diagram of the tested original two-factor SPQ-C model. SPQ1-SPQ11 = Sports Performance Questionnaire items 1–11.

The global fit indices were mixed but broadly adequate. For the original two-factor model, CFI (0.967), TLI (0.958), and SRMR (0.027) met commonly used criteria, whereas RMSEA (0.068, 90% CI [0.055, 0.081]) was slightly above the stringent good-fit threshold of 0.06 and therefore fell within a reasonable-to-mediocre range. The unidimensional model showed a very similar pattern of fit, with CFI = 0.968, TLI = 0.959, SRMR = 0.027, and RMSEA = 0.067, 90% CI [0.054, 0.080], which also fell within a reasonable-to-mediocre range (Table 3). Therefore, model selection was not based solely on global fit indices, but primarily on the discriminant validity failure of the original two-factor model. The original two-factor structure was not retained because the correlation between Persistence and Skill was extremely high (r = 0.996), indicating empirical indistinguishability between the two latent factors. Accordingly, the unidimensional model was retained as the final SPQ-C structure. In the final model, all standardized factor loadings exceeded the minimum threshold of 0.40 and ranged from 0.609 to 0.841 (Figure 1; Table 4). For transparency, the standardized factor loadings of the tested original two-factor model are provided in Figure 2 and Table 5, but this model was not retained due to lack of discriminant validity. These results are reported only to document the model-testing process and to explain why the original two-factor structure was not adopted as the final SPQ-C structure.

**Table 3:** CFA fit indices for the SPQ-C (original two-factor and final unidimensional models).

Model	RMSEA (90% CI)	CFI	TLI	SRMR
Tested original two-factor	0.068 (0.055, 0.081)	0.967	0.958	0.027
Retained unidimensional	0.067 (0.054, 0.080)	0.968	0.959	0.027

RMSEA, Root Mean Square Error of Approximation; CFI, Comparative Fit Index; TLI, Tucker-Lewis Index; SRMR, Standardized Root Mean Square Residual.

**Table 4:** Standardized factor loadings of the retained unidimensional SPQ-C model.

Factors/items	Factor loading	Cronbach’s alpha
Sport performance		0.932
SPQ1	0.653	
SPQ2	0.609	
SPQ3	0.630	
SPQ4	0.841	
SPQ5	0.825	
SPQ6	0.805	
SPQ7	0.798	
SPQ8	0.771	
SPQ9	0.785	
SPQ10	0.777	
SPQ11	0.686	

**Table 5:** Standardized factor loadings of the tested original two-factor SPQ-C model.

Factors/items	Factor loading	Cronbach’s alpha
Persistence		0.905
SPQ1	0.653	
SPQ2	0.609	
SPQ3	0.630	
SPQ4	0.842	
SPQ6	0.806	
SPQ7	0.798	
SPQ8	0.771	
SPQ9	0.785	
Skill		0.809
SPQ5	0.827	
SPQ10	0.779	
SPQ11	0.688	

### Convergent and discriminant validity

The original two-factor model was not retained because it failed to demonstrate discriminant validity. Specifically, the correlation between the Persistence and Skill factors was extremely high (r = 0.996, *p <* 0.05), indicating that the two latent factors were empirically indistinguishable in the present coach-rated sample. This result failed to satisfy the Fornell–Larcker criterion for discriminant validity (Fornell and Larcker, 1981). Therefore, the subsequent validity and reliability interpretation focused on the retained unidimensional model.

For the final unidimensional model, all standardized factor loadings exceeded the minimum acceptable threshold and ranged from 0.609 to 0.841. The model demonstrated excellent internal consistency (Cronbach’s *α* = 0.932) and composite reliability (CR = 0.932). The AVE value was 0.559, exceeding the recommended threshold of 0.50 and supporting convergent validity. Because the retained model included only one latent construct, discriminant validity between latent factors was no longer applicable (Table 6).

**Table 6:** Reliability and validity evidence for the tested original two-factor and retained unidimensional SPQ-C models.

Tested original two-factor	CR	AVE	Persistence	Skill
Persistence	0.906	0.550	0.742	
Skill	0.810	0.588	0.996*	0.767

Retained unidimensional	CR	AVE
Sport Performance	0.932	0.559

*p < 0.05; CR, Composite Reliability; AVE, Average Variance Extracted. Diagonal values for the tested original two-factor model represent the square root of AVE. The original two-factor model was not retained because the correlation between Persistence and Skill exceeded the discriminant validity criterion. The retained unidimensional model contains only one latent construct; therefore, discriminant validity between latent factors is not applicable.

### Test–retest reliability

The SPQ-C showed high preliminary test–retest reliability in the retest subsample. The ICC for the sport performance dimension was 0.935, indicating high temporal stability among the 50 players rated by coaches from one elite football school over a two-week interval. However, because this subsample was relatively small and drawn from a single elite institution, the ICC should be interpreted as preliminary evidence rather than definitive evidence of temporal stability across all Chinese youth football contexts. Table 7 presents the ICC value for the SPQ-C.

**Table 7:** Intraclass correlation coefficients (ICC) for the SPQ-C.

Dimension	ICC
Sport Performance	0.935

ICC, Intraclass Correlation Coefficient.

## Discussion

The present investigation’s primary contribution lies in showing that the SPQ-C was more appropriately represented as a unidimensional construct than as the original two-factor structure proposed by Sivrić (2021). This finding aligns with broader patterns observed in cross-cultural adaptation studies, where cultural factors significantly influence construct conceptualization and factorial structure (Quansah et al., 2024). The extremely high inter-factor correlation observed between Persistence and Skill suggests that, in the present Chinese coach-rating context, the performance indicators were represented as a unified construct rather than as empirically separable dimensions.

This structural preference resonates with previous cross-cultural validation studies in sports psychology that have identified cultural variations in psychological construct organization. For instance, the cross-cultural adaptation of the Sport Emotion Questionnaire across African countries revealed similar structural modifications when instruments were adapted for non-Western populations (Quansah et al., 2024) Similarly, the validation of psychological assessment tools in Chinese contexts has consistently demonstrated the need for factorial structure reconsideration to align with cultural cognitive frameworks (Wang et al., 2024). The decision to adopt the unidimensional structure was primarily driven by the violation of discriminant validity criteria, as evidenced by the inter-factor correlation of 0.996 substantially exceeding the recommended threshold of 0.85. This finding suggests that, in the present Chinese coach-rating context, the assessed performance characteristics were represented as components of a unified construct rather than as empirically distinct dimensions.

An additional item-level issue concerns SPQ10, which assessed talent and showed the lowest mean score and the highest proportion of neutral coach ratings. This pattern may indicate that talent is a more inferential and less directly observable performance characteristic than behavioral indicators such as cooperation, self-discipline, or tactical adherence. Coaches may therefore be more cautious when assigning high or low ratings on this item, particularly for early adolescent players whose developmental potential is still emerging. However, SPQ10 showed a strong standardized loading in the final unidimensional model, suggesting that it remained psychometrically meaningful within the overall sport performance construct. Therefore, SPQ10 was retained in the SPQ-C. Future research should further examine this item using cognitive interviews with coaches, item response analysis, or qualitative feedback to determine whether its interpretation is consistent across different coaching contexts and competitive levels.

The theoretical implications of adopting a unidimensional structure extend beyond mere statistical considerations. As noted by Behnke et al. (2019) in their cross-cultural validation of the Sport Mental Training Questionnaire, cultural context fundamentally shapes how athletes conceptualize and organize their performance-related attributes. This pattern may also be related to broader cultural and contextual features of performance evaluation; however, this interpretation should be treated cautiously because the present data were based on coach ratings rather than direct assessment of athletes’ own conceptualizations.

The successful validation of the SPQ-C directly addresses the measurement challenges identified in contemporary football performance research. As highlighted by McAuley et al. (2022), traditional ordinal classification systems have proven inadequate for capturing the nuanced aspects of player development and performance variability. The SPQ-C provides a standardized, multidimensional assessment approach that transcends these limitations by incorporating both psychological attributes and skill-based competencies within a unified measurement framework.

This advancement is particularly significant given the complexity of football performance assessment noted by Bonney et al. (2019), who emphasized the sport’s demands for integration across physical, technical, tactical, and psychological domains. The SPQ-C’s comprehensive coverage of these dimensions, combined with its cultural appropriateness for Chinese populations, represents a substantial improvement over existing assessment methodologies that often fail to capture performance variability across different contexts (Lepschy et al., 2018).

Furthermore, the instrument’s focus on coach perceptions aligns with recommendations from Musculus and Lobinger (2018) regarding the critical importance of expert assessment in youth football evaluation. Coaches, as noted by these researchers, possess unique insights into on-field performance characteristics and success-related traits that may not be captured through self-report measures or objective performance metrics alone.

The validation of the SPQ-C occurs within the broader context of China’s ambitious football development initiatives, particularly the comprehensive national reform plan initiated in 2015 that outlined ambitious goals for youth football participation and talent development (General Office of the State Council, 2015). Recent research has consistently highlighted the lack of standardized assessment tools as a significant barrier to effective talent identification and development in Chinese youth football (Yang, 2025).

The SPQ-C addresses this critical gap by providing a culturally appropriate, psychometrically sound instrument that can support systematic talent identification and development processes. This contribution is particularly timely given the establishment of over 32,000 football specialty schools across China, which currently lack systematic evaluation methods for assessing youth football talent. The fragmented approaches across different governing bodies identified by Gündoğan (2024) further underscore the need for standardized assessment tools that can facilitate consistent performance evaluation and talent identification.

Recent studies have emphasized the importance of developing culturally appropriate evaluation methods that can effectively assess both technical and psychological aspects of football performance in Chinese contexts (Wang et al., 2024). The SPQ-C fulfills this requirement by maintaining conceptual equivalence with international standards while adapting to Chinese cultural frameworks, thereby supporting the integration of evidence-based assessment tools into Chinese youth football programs as advocated by Yang (2025).

The validated SPQ-C provides immediate practical benefits for Chinese youth football practitioners, including coaches, sport psychologists, and talent development specialists. The instrument’s unidimensional structure simplifies interpretation and application while maintaining comprehensive assessment coverage, which is particularly important in applied settings where practitioners require efficient yet thorough evaluation tools. Beyond individual assessment, the SPQ-C offers valuable applications for program evaluation and development planning, enabling youth football programs to identify areas requiring targeted intervention, monitor athlete development trajectories, and evaluate training methodology effectiveness.

Future research should explore several important directions to maximize the instrument’s utility and theoretical contributions. Investigation of predictive validity through correlation with objective performance outcomes would strengthen the instrument’s theoretical foundation and practical utility, while examination of measurement invariance across gender, age groups, and competitive levels would establish its appropriateness for diverse athlete populations within Chinese youth football. Additionally, integration of the SPQ-C with complementary assessment tools targeting motivation, goal orientation, and cultural values could provide comprehensive understanding of factors influencing athletic success in Chinese contexts, aligning with contemporary trends toward holistic athlete assessment and development.

### Limitations and future research

Several methodological limitations warrant acknowledgment for future research consideration. The exclusive focus on urban areas in Shandong Province limits the generalizability of the findings to other Chinese regions with different cultural, economic, educational, and football development characteristics. Therefore, the present findings should be interpreted as preliminary validation evidence for an urban Shandong youth football context rather than as national normative evidence for all Chinese adolescent football players. Future validation studies should include samples from rural areas, multiple provinces, different school types, and diverse competitive levels to examine whether the unidimensional structure remains stable across broader Chinese youth football populations. The reliance on coach ratings, while appropriate for the SPQ’s design, represents a single-source measurement approach that may introduce perspective limitations, suggesting the need for multi-source validation incorporating athlete self-reports, peer evaluations, and objective performance metrics. Finally, the test–retest analysis was based on a relatively small subsample of 50 players from a single elite football school. Although the ICC was high, this result may be influenced by the relatively homogeneous training environment, coaching standards, and player characteristics within that institution. Therefore, the test–retest findings should be regarded as preliminary. Future studies should examine temporal stability using larger retest samples drawn from multiple schools and competitive levels. In addition, longitudinal studies tracking athletes across multiple seasons would provide valuable evidence regarding measurement stability, sensitivity to performance changes, and the utility of the SPQ-C for monitoring athlete development over time.

## Conclusion

This study validated the Chinese adaptation of the Sports Performance Questionnaire among adolescent football players from urban areas of Shandong Province. The original two-factor structure was not retained because Persistence and Skill were empirically indistinguishable in the present coach-rated sample. The retained unidimensional SPQ-C showed satisfactory reliability and validity evidence and may serve as a culturally appropriate coach-rated tool for assessing performance-related characteristics in Chinese youth football contexts. Further validation is needed in more geographically diverse samples, rural settings, different competitive levels, and larger multi-school test–retest samples.

## Data Availability

The raw data supporting the conclusions of this article will be made available by the authors, without undue reservation.
