# Supplementary material for: Cross-cultural validation of the sports performance questionnaire in Chinese adolescent football players: a confirmatory factor analysis study
Source: Front Psychol. 2026 Jul 23;17:1837560. doi: 10.3389/fpsyg.2026.1837560 (PMC13441716; doi:10.3389/fpsyg.2026.1837560)
Supplement: Supplementary file 1 [file Supplementary_file_1.pdf]

Appendix

Football Success Questionnaire

Football player's first and last name:

The following pages list the usual characteristics of football players. Use the instructions below to assess the extent to which these characteristics apply to each soccer player . Your responses will be kept confidential and will only be used for further analysis.

illustrate:
On a scale from 1 (not at all) to 5 (exactly) , evaluate how well a soccer player performs on the characteristics described below?

Competitiveness in a competitive environment	1	2	3	4	5
confidence in competition	1	2	3	4	5
desire for progress	1	2	3	4	5
cooperate	1	2	3	4	5
On-site communication	1	2	3	4	5
self-criticism	1	2	3	4	5
coping with stressful situations	1	2	3	4	5
self-discipline	1	2	3	4	5
follow tactical strategy	1	2	3	4	5
talent	1	2	3	4	5
creativity	1	2	3	4	5

Football player's position:____________

足球运动表现测试

在从 1（根本不符合）到 5（完全符合）的范围内，评估你在以下所述特征中的表现如何？

1 完全不符合 2 不太符合　3 无法确定　4 较为符合　5 完全符合

题目	1	2	3	4	5
1.　竞争环境下的竞争力					
2.　竞赛中的自信					
3.　进步的渴望					
4.　合作					
5.　现场沟通					
6.　自我批评					
7.　应对压力情况					
8.　自律					
9.　遵循战术战略要求					
10.　天赋					
11.　创造力					
